# Regional Infoveillance of COVID-19 Case Rates: Analysis of Search-Engine Query Patterns

**DOI:** 10.2196/19483

**Published:** 2020-07-30

**Authors:** Henry C Cousins, Clara C Cousins, Alon Harris, Louis R Pasquale

**Affiliations:** 1 Department of Genetics Stanford School of Medicine Stanford, CA United States; 2 Department of Biological Engineering Massachusetts Institute of Technology Cambridge, MA United States; 3 Department of Data Sciences Dana-Farber Cancer Institute, Harvard TH Chan School of Public Health Boston, MA United States; 4 Center for Functional Cancer Epigenetics Dana-Farber Cancer Institute Boston, MA United States; 5 Department of Ophthalmology Icahn School of Medicine at Mount Sinai New York, NY United States

**Keywords:** epidemiology, infoveillance, COVID-19, internet activity, Google Trends, infectious disease, surveillance, public health

## Abstract

**Background:**

Timely allocation of medical resources for coronavirus disease (COVID-19) requires early detection of regional outbreaks. Internet browsing data may predict case outbreaks in local populations that are yet to be confirmed.

**Objective:**

We investigated whether search-engine query patterns can help to predict COVID-19 case rates at the state and metropolitan area levels in the United States.

**Methods:**

We used regional confirmed case data from the New York Times and Google Trends results from 50 states and 166 county-based designated market areas (DMA). We identified search terms whose activity precedes and correlates with confirmed case rates at the national level. We used univariate regression to construct a composite explanatory variable based on best-fitting search queries offset by temporal lags. We measured the raw and z-transformed Pearson correlation and root-mean-square error (RMSE) of the explanatory variable with out-of-sample case rate data at the state and DMA levels.

**Results:**

Predictions were highly correlated with confirmed case rates at the state (mean *r*=0.69, 95% CI 0.51-0.81; median RMSE 1.27, IQR 1.48) and DMA levels (mean *r*=0.51, 95% CI 0.39-0.61; median RMSE 4.38, IQR 1.80), using search data available up to 10 days prior to confirmed case rates. They fit case-rate activity in 49 of 50 states and in 103 of 166 DMA at a significance level of .05.

**Conclusions:**

Identifiable patterns in search query activity may help to predict emerging regional outbreaks of COVID-19, although they remain vulnerable to stochastic changes in search intensity.

## Introduction

Early detection of regional coronavirus disease (COVID-19) outbreaks is essential for efficient medical resource allocation, public health messaging, and implementation of infection prevention and control strategies [1]. It is particularly important given the probability of future waves of COVID-19 cases and the difficulty of applying traditional epidemiological forecasting models in areas with low case levels [2,3]. However, laboratory testing capacity is limited, and confirmed case reports lag behind underlying infections, decreasing their predictive capacity in the early days of an outbreak or resurgence.

Internet browsing data, such as search-engine query results, can provide a real-time indication of symptoms in a population and have been used extensively to predict and model outbreaks like influenza and dengue [4-7]. Such methods generally assume that specific and detectable patterns in internet behavior, such as search trends or social media postings, reflect health-seeking behavior in real time at the population level. Forecasting models based on search queries, such as Google Flu Trends, have shown predictive value without direct reliance on formal case reports, although historical inaccuracies mean that they can only supplement, not replace, traditional forecasting methodologies based on confirmed cases [8-10].

COVID-19 case rates display significant regional heterogeneity that requires locally tailored containment strategies. Google search trends, encompassing a majority of internet queries in the United States and publicly available through Google Trends (GT), provide a powerful resource for systematic comparison of browsing behavior between US regions. We hypothesized that keyword libraries could be screened for specific terms whose aggregate activity would reflect regional differences in COVID-19 case rates, as has been demonstrated for influenza [4]. While several studies have previously attempted to model the COVID-19 pandemic using search query data, such attempts have largely focused on specific regions, like Taiwan and Iran, and a limited number of individually selected search terms [11-14]. We explored the potential of large-scale, publicly accessible search query data to signal new COVID-19 cases at the state and metropolitan-area levels in the United States.

## Methods

### Data Collection and Processing

We obtained confirmed case data for US states and counties from the New York Times (NYT) data set from January 21, the date of the first confirmed US case, to April 2, 2020, comprising county-specific, lab-confirmed COVID-19 case reports compiled daily from local and state health authorities [15]. We used the NYT data set because of both its inclusion of county-level case geotags and its strong correlation with other case tracking sources [16]. Next, we used GT to compile a library of 463 unique search queries and their associated daily activity levels over the same time period. Library terms were automatically retrieved based on the likelihood of user association with a set of prespecified coronavirus-related seed terms (Multimedia Appendix 1), using the GT “Related Queries” function.

We compared the z-transformed correlation of each query’s search activity with an in-sample data set comprising daily confirmed national cases rates for days through March 10 with >100 new cases per day. Each query’s search activity was offset by temporal lags of between 0 and 14 days, generating a list of best-fitting queries and their associated optimal lag times. To focus on terms with early predictive power, we excluded queries whose optimal lag was less than 9 days. We selected the five best-fitting queries and constructed a single explanatory variable by summing the lag-adjusted, relative activity levels of each query. Finally, we linearly fit the explanatory variable to national data through March 10 to generate a single scalar coefficient.

### Data Analysis

We measured the correlation of state-specific activity levels for our explanatory variable with daily reported case levels in individual states using out-of-sample data from March 11 through April 2. We also measured how well the explanatory variable explained out-of-sample case rates in 166 designated market areas (DMA), which are collections of approximately 15 counties each constituting the highest-resolution regional data available on GT. Means and confidence intervals for correlation coefficients were calculated using the inverse z-transformation of the averaged z-transformed coefficients. The strength of model predictions over time was measured using a partial correlation of first confirmed case dates with z-transformed correlation coefficients in all regions with >100 cases, controlling for regional population. We used root-mean-square error (RMSE) as an additional measure of model performance. Model predictions were adjusted for regional population and internet access [17]. All data were anonymous, and the study protocol was approved by the institutional review board of the Icahn School of Medicine at Mount Sinai.

## Results

### Search Query Characteristics

Queries incorporated into the final explanatory variable were highly correlated with national case data, with correlation coefficients ranging from 0.996 to 0.999 on the in-sample data. The optimal temporal lags for incorporated queries were from 11 to 12 days, indicating a prediction horizon of up to 10 days (assuming that a day’s full GT query results become available on the subsequent day). The final variable, the linear sum of weighted, lag-adjusted activity levels for the five best-fitting terms from the 463-term library, fit the in-sample data with a correlation of 0.998.

Characteristics of additional screened queries validated our methodology. For instance, acute topics like medical care and testing had smaller associated lag times with confirmed case rates, as would be expected for urgent inquiries (Table 1). Queries unrelated to COVID-19 had correspondingly weaker correlations with the observed data. The best-fitting category of queries was “COVID-19 guidance,” which included terms related to coronavirus-specific medical advice from health authorities. Relative levels of search activity had no significant effect on fit with case data.

**Table 1:** Characteristics of query topics screened for fit with coronavirus disease (COVID-19) case data.

Search query category	Unique queries^a^, n (%)	Correlation with national case rate^b^, mean	Associated lag time (days)^c^, mean	Activity weighting^d^
COVID-19 guidance	32 (6.9)	0.96	9.1	0.38
COVID-19 news	57 (12.3)	0.96	8.3	1.00
COVID-19 symptoms	91 (19.7)	0.94	8.9	0.41
Medical treatments	34 (7.3)	0.93	10.1	0.31
COVID-19 testing	58 (12.5)	0.89	5.4	0.11
Medical care	33 (7.1)	0.89	7.2	0.60
Nonspecific symptoms	62 (13.4)	0.89	6.8	0.57
Economic effects	28 (6.0)	0.86	5.9	0.12
Unrelated to illness	51 (11.0)	0.86	6.6	0.76
Symptoms of other illnesses	17 (3.7)	0.84	8.3	0.77

^a^Number of queries of each type in the query library (eg, the category “COVID-19 testing” would include the specific query “coronavirus test near me,” and the category “nonspecific symptoms” would include the query “cough”).

^b^Expressed as the inverse z-transformation of the averaged z-transformed correlations with in-sample national data.

^c^Mean lag time between best-fitting query activity and confirmed case rate, in days.

^d^Relative mean search activity levels, normalized.

### Regional Case-Rate Predictions

**Figure 1:**
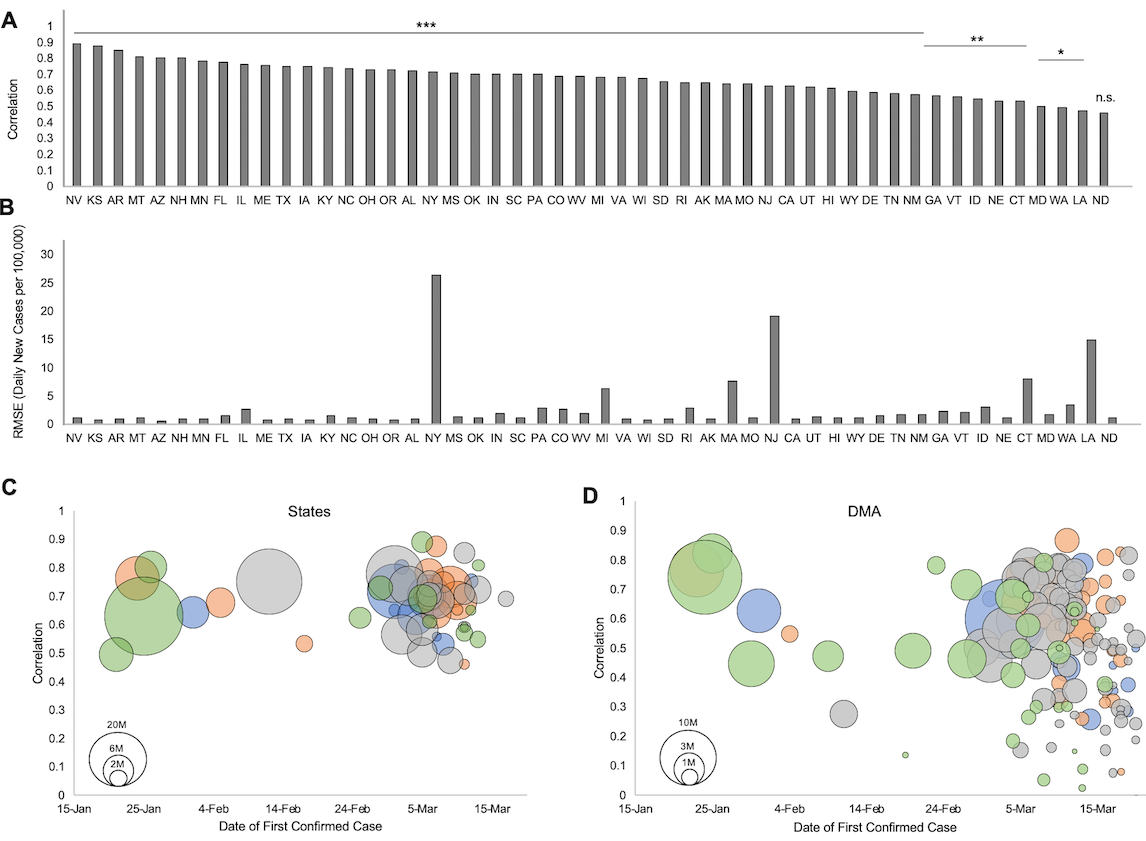
Correlation of query predictions with regional coronavirus disease (COVID-19) confirmed case rates. (A) Correlation of predicted case rates with actual case rates for the 50 states. Values are Pearson correlation coefficients. * indicates significance at α=.05; ** at α=.01; *** at α=.005. (B) Root-mean-square error (RMSE) between predicted case rates and actual case rates for the 50 states, in units of daily new cases per 100,000 population. (C) Prediction correlations at the state level do not depend on outbreak timing, as measured by the date of the first confirmed case. Circle size indicates the relative population of the state. Color indicates US census-designated region (blue: Northeast; orange: Midwest; gray: South; green: West). (D) Prediction correlations at the designated market area (DMA) level do not depend on outbreak timing, as measured by the date of the first confirmed case. Circle size indicates the relative population of DMA. Color indicates the US census-designated region, as described. n.s.: not significant.

The query-based predictions fit well with out-of-sample case rate data at the national level, with a correlation of 0.84 (*P*<.001) for out-of-sample data and 0.83 (*P*<.001) for all available data. The predictions were also well correlated at the state level in nearly all cases, fitting case data in 49 of 50 states at a significance level of α=.05 and 41 of 50 states at α=.005 (mean *r*=0.69; 95% CI 0.51-0.81; Figure 1A; Multimedia Appendix 2). RMSE was less than 4 cases per 100,000 residents for model predictions in 44 of 50 states (median 1.27; IQR 1.48; Figure 1B).

**Figure 2:**
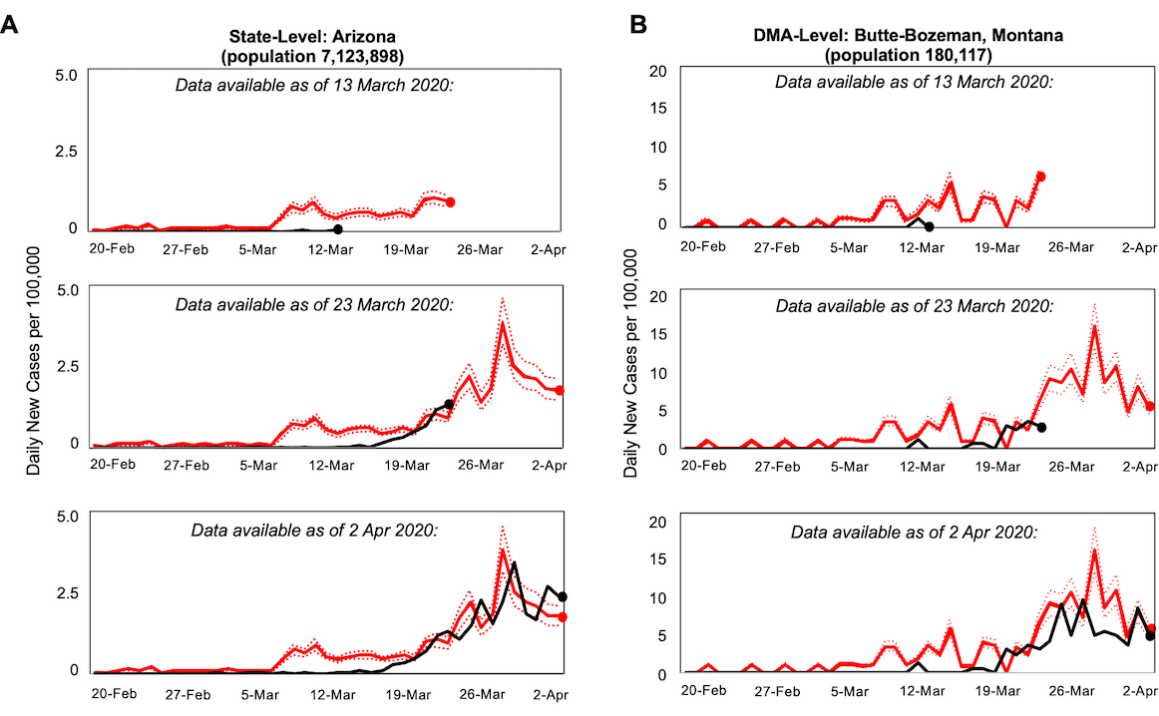
Correlation of query predictions (red) with regional coronavirus disease (COVID-19) case rates (black) at the state and designated market area (DMA) levels, February 20 to April 2, 2020. (A) Comparison of predicted case rates (red) with actual case rates (black) at the state level, with Arizona shown as an example. Dashed lines indicate 95% CIs. (B) Comparison at the DMA level, with the Butte-Bozeman area shown as an example of predictions in a low-population region.

At the DMA level, the query-based predictions fit with daily case data for 62% (103/166) of regions at α=.05, or 79% (84/107) excluding DMA with fewer than 100 cases (mean *r* for all DMA=0.51; 95% CI 0.39-0.61; Multimedia Appendix 3). RMSE was slightly higher for DMA-level compared to state-level predictions but was less than 7 for 92% (152/166) of DMA (median 4.38; IQR 1.80). Furthermore, at both the state and DMA levels, the strength of the correlation was not significantly associated with the date of a region’s first confirmed case (*P*=.51 for states and *P*=.71 for DMA for partial correlations in regions with >100 cases, controlling for population), suggesting that predictive search behaviors may precede new cases regardless of the timing of a regional outbreak (Figure 1C and D). The explanatory variable consistently produced well-fitting predictions with data available 10 days in advance of predicted new case activity (Figure 2), even in regions where fewer than 100 new cases were confirmed per day.

## Discussion

These data suggest that specific patterns of internet search behavior, which can be curated automatically from libraries of search terms, precede and correlate with regional case rates of COVID-19. Such patterns, which we captured using a single explanatory variable, remain correlated with case rates in regions with a broad range of populations, locations, and outbreak times, making aggregate search trends a useful tool for estimating regional COVID-19 outbreaks in the days preceding confirmed case reports. Correlation strengths were not significantly associated with the date of onset of regional outbreaks, making it unlikely that a single national event, such as a press release, could explain the strength of model predictions in all regions. Furthermore, search queries explicitly related to COVID-19 have more predictive power than unrelated keywords, and acute queries, such as those concerning testing or medical care, have smaller associated lag times.

Taken together, these results suggest that systematic screening of key term libraries can identify search queries reflecting real-time health-seeking behaviors at the regional level, expanding the suite of “infoveillance” methods that may assist in monitoring COVID-19 cases. This type of approach does not directly depend on either regional testing capacity or local media reports, making it particularly relevant in areas with small populations, limited medical infrastructure, or low case numbers. Such information can supplement traditional epidemiological approaches, such as estimates based on a compartmental framework, to guide community health interventions in the early days of an outbreak.

Several aspects of query-based approaches to case estimation, such as this work, must be further characterized for COVID-19. First, while correlations were statistically strong across most US regions, elevated RMSE indicated lower accuracy for predictions in the New York City and New Orleans areas, both regions with major outbreaks. However, comparable losses in accuracy were not observed for other major outbreak sites, such as Philadelphia, Los Angeles, or Chicago. This may reflect region-specific differences in both internet browsing behavior and patterns of community infection and may be a limitation of query-based models using fixed terms. As evidenced by previous attempts to predict influenza outbreaks based on search data, browsing behavior will also likely change as public understanding evolves over the course of disease spread [18]. Therefore, search-term relevance is likely to vary with time, which may require continuous supplementation or reselection of query terms to ensure representativeness of current population behaviors. Furthermore, although we generally observed strong historical correlations, query-based models must also be monitored for sudden changes in COVID-related query activity due to external events, such as unrelated news reports. Such distortions would be particularly important in regions with limited internet access. Future models incorporating learning and real-time updating of region-specific search terms may improve query-based prediction efforts for future COVID-19 outbreaks.

## Appendix

Multimedia Appendix 1 Seed query terms.

Multimedia Appendix 2 Performance of model predictions for individual states.

Multimedia Appendix 3 Performance of model predictions for individual designated market areas (DMA).

## Abbreviations

COVID-19: coronavirus disease
DMA: designated market area
GT: Google Trends
NYT: New York Times
RMSE: root-mean-square error
