# Supplementary material for: Regional Infoveillance of COVID-19 Case Rates: Analysis of Search-Engine Query Patterns
Source: J Med Internet Res. 2020 Jul 30;22(7):e19483. doi: 10.2196/19483 (PMC7394521; doi:10.2196/19483)
Supplement: Multimedia Appendix 1 [file jmir_v22i7e19483_app1.docx]

Multimedia Appendix 1. Seed query terms.

The screening library of 463 search queries was obtained using the GT “Related Queries” function on an initial bank of 23 coronavirus-related terms.

|  | **Seed Term** |
| --- | --- |
| 1 | 'am i sick' |
| 2 | 'cdc' |
| 3 | 'corona virus near me' |
| 4 | 'coronavirus doctor' |
| 5 | 'coronavirus help' |
| 6 | 'coronavirus hospital' |
| 7 | 'coronavirus symptoms' |
| 8 | 'coronavirus testing' |
| 9 | 'coronavirus treatment' |
| 10 | 'coronavirus vs flu symptoms' |
| 11 | 'cough' |
| 12 | 'covid 19 symptoms' |
| 13 | 'covid 19 testing' |
| 14 | 'do i have coronavirus' |
| 15 | 'doctor near me' |
| 16 | 'fever' |
| 17 | 'headache' |
| 18 | 'hospital near me' |
| 19 | 'insurance coronavirus' |
| 20 | 'sick' |
| 21 | 'sore throat' |
| 22 | 'symptoms coronavirus 2020' |
| 23 | 'testing' |
