# Supplementary material for: Regional Infoveillance of COVID-19 Case Rates: Analysis of Search-Engine Query Patterns
Source: J Med Internet Res. 2020 Jul 30;22(7):e19483. doi: 10.2196/19483 (PMC7394521; doi:10.2196/19483)
Supplement: Multimedia Appendix 2 [file jmir_v22i7e19483_app2.docx]

Multimedia Appendix 2. Performance of model predictions for individual states.

The model was evaluated on out-of-sample data from March 11 – April 2 as described in Methods. r: untransformed Pearson correlation coefficient; *P*: p-value associated with r; RMSE: root-mean-square error in units of daily new cases per 100,000 population.

| **﻿State** | **r** | ***P*** | **RMSE** |
| --- | --- | --- | --- |
| AK | .65 | <.001 | 1.01 |
| AL | .72 | <.001 | .97 |
| AR | .85 | <.001 | .90 |
| AZ | .80 | <.001 | .62 |
| CA | .63 | <.001 | .90 |
| CO | .69 | <.001 | 2.61 |
| CT | .53 | .01 | 8.13 |
| DC | .05 | .81 | 6.00 |
| DE | .59 | <.001 | 1.53 |
| FL | .78 | <.001 | 1.55 |
| GA | .56 | .01 | 2.33 |
| HI | .61 | <.001 | 1.21 |
| IA | .75 | <.001 | .83 |
| ID | .55 | .01 | 3.05 |
| IL | .76 | <.001 | 2.73 |
| IN | .71 | <.001 | 2.02 |
| KS | .88 | <.001 | .76 |
| KY | .74 | <.001 | 1.46 |
| LA | .47 | .02 | 14.89 |
| MA | .64 | <.001 | 7.61 |
| MD | .50 | .02 | 1.83 |
| ME | .75 | <.001 | .77 |
| MI | .68 | <.001 | 6.33 |
| MN | .78 | <.001 | .94 |
| MO | .64 | <.001 | 1.22 |
| MS | .71 | <.001 | 1.28 |
| MT | .81 | <.001 | 1.13 |
| NC | .74 | <.001 | 1.11 |
| ND | .46 | .05 | 1.10 |
| NE | .53 | .01 | 1.26 |
| NH | .80 | <.001 | 1.02 |
| NJ | .63 | <.001 | 19.09 |
| NM | .57 | <.001 | 1.67 |
| NV | .89 | <.001 | 1.14 |
| NY | .72 | <.001 | 26.35 |
| OH | .73 | <.001 | .91 |
| OK | .71 | <.001 | 1.22 |
| OR | .73 | <.001 | .77 |
| PA | .70 | <.001 | 2.82 |
| RI | .65 | <.001 | 2.87 |
| SC | .70 | <.001 | 1.11 |
| SD | .65 | <.001 | 1.01 |
| TN | .58 | <.001 | 1.69 |
| TX | .75 | <.001 | .91 |
| UT | .62 | <.001 | 1.27 |
| VA | .68 | <.001 | .93 |
| VT | .56 | .01 | 2.19 |
| WA | .49 | .02 | 3.46 |
| WI | .68 | <.001 | .84 |
| WV | .69 | <.001 | 1.91 |
| WY | .59 | <.001 | 1.27 |
