# Supplementary material for: Regional Infoveillance of COVID-19 Case Rates: Analysis of Search-Engine Query Patterns
Source: J Med Internet Res. 2020 Jul 30;22(7):e19483. doi: 10.2196/19483 (PMC7394521; doi:10.2196/19483)
Supplement: Multimedia Appendix 3 [file jmir_v22i7e19483_app3.docx]

Multimedia Appendix 3. Performance of model predictions for individual designated market areas (DMA).

The model was evaluated on out-of-sample data from March 11 – April 2 as described in Methods. r: untransformed Pearson correlation coefficient; *P*: p-value associated with r; RMSE: root-mean-square error in units of daily new cases per 100,000 population.

| **DMA ID** | **Description** | **r** | ***P*** | **RMSE** |
| --- | --- | --- | --- | --- |
| US-AK-743 | ANCHORAGE | .30 | .16 | 5.59 |
| US-AK-745 | FAIRBANKS | .57 | .01 | 3.51 |
| US-AK-747 | JUNEAU | .20 | .53 | 5.20 |
| US-AL-522 | COLUMBUS | .47 | .04 | 4.94 |
| US-AL-606 | DOTHAN | -.11 | .68 | 6.80 |
| US-AL-630 | BIRMINGHAM | .63 | <.001 | 2.65 |
| US-AL-691 | HUNTSVILLE-DECATUR-FLORENCE | .72 | <.001 | 5.22 |
| US-AR-640 | MEMPHIS | .65 | <.001 | 2.21 |
| US-AR-670 | FORT SMITH-FAYETTEVILLE-SPRINGDALE | .64 | .01 | 6.12 |
| US-AR-693 | LITTLE ROCK-PINE BLUFF | .67 | <.001 | 3.61 |
| US-AR-734 | JONESBORO | .29 | .27 | 5.96 |
| US-AZ-753 | PHOENIX | .82 | <.001 | 3.28 |
| US-AZ-789 | TUCSON-SIERRA VISTA | .79 | <.001 | 4.23 |
| US-CA-800 | BAKERSFIELD | .38 | .12 | 2.92 |
| US-CA-802 | EUREKA | .14 | .63 | 4.97 |
| US-CA-803 | LOS ANGELES | .74 | <.001 | 3.28 |
| US-CA-807 | SAN FRANCISCO-OAKLAND-SAN JOSE | .45 | .03 | 3.29 |
| US-CA-811 | RENO | .18 | .40 | 5.77 |
| US-CA-813 | MEDFORD-KLAMATH FALLS | .67 | .01 | 3.60 |
| US-CA-825 | SAN DIEGO | .47 | .02 | 3.15 |
| US-CA-828 | MONTEREY-SALINAS | .27 | .23 | 5.23 |
| US-CA-862 | SACRAMENTO-STOCKTON-MODESTO | .49 | .02 | 4.41 |
| US-CA-866 | FRESNO-VISALIA | .58 | <.001 | 3.73 |
| US-CA-868 | CHICO-REDDING | .05 | .86 | 5.38 |
| US-CO-751 | DENVER | .68 | <.001 | 2.31 |
| US-CO-752 | COLORADO SPRINGS-PUEBLO | .70 | <.001 | 3.84 |
| US-CO-773 | GRAND JUNCTION-MONTROSE | .03 | .91 | 4.74 |
| US-CT-533 | HARTFORD-NEW HAVEN | .43 | .04 | 3.93 |
| US-FL-528 | MIAMI-FORT LAUDERDALE | .74 | <.001 | 3.16 |
| US-FL-530 | TALLAHASSEE-THOMASVILLE | .34 | .14 | 3.89 |
| US-FL-534 | ORLANDO-DAYTONA BEACH-MELBOURNE | .78 | <.001 | 3.91 |
| US-FL-539 | TAMPA-ST PETERSBURG-SARASOTA | .50 | .01 | 3.10 |
| US-FL-561 | JACKSONVILLE | .68 | <.001 | 2.72 |
| US-FL-571 | FORT MYERS-NAPLES | .77 | <.001 | 2.48 |
| US-FL-592 | GAINESVILLE | .16 | .46 | 3.56 |
| US-FL-656 | PANAMA CITY | .49 | .04 | 4.94 |
| US-FL-686 | MOBILE-PENSACOLA | .71 | <.001 | 3.40 |
| US-GA-520 | AUGUSTA | .36 | .14 | 4.41 |
| US-GA-524 | ATLANTA | .46 | .03 | 3.35 |
| US-GA-525 | ALBANY | .45 | .05 | 16.87 |
| US-GA-575 | CHATTANOOGA | .67 | <.001 | 3.54 |
| US-HI-744 | HONOLULU | .50 | .02 | 4.67 |
| US-IA-637 | CEDAR RAPIDS-WATERLOO-DUBUQUE | .78 | <.001 | 2.80 |
| US-IA-679 | DES MOINES-AMES | .62 | <.001 | 3.42 |
| US-IA-682 | DAVENPORT-ROCK ISLAND-MOLINE | .53 | .02 | 4.22 |
| US-ID-757 | BOISE | .63 | <.001 | 3.00 |
| US-ID-758 | IDAHO FALLS-POCATELLO | .09 | .70 | 5.22 |
| US-ID-881 | SPOKANE | .78 | <.001 | 3.73 |
| US-IL-602 | CHICAGO | .77 | <.001 | 2.45 |
| US-IL-632 | PADUCAH-CAPE GIRARDEAU-HARRISBURG | .81 | <.001 | 4.82 |
| US-IL-648 | CHAMPAIGN-SPRINGFIELD-DECATUR | .67 | <.001 | 4.91 |
| US-IL-649 | EVANSVILLE | .46 | .07 | 4.02 |
| US-IL-675 | PEORIA-BLOOMINGTON | .26 | .25 | 3.64 |
| US-IN-509 | FORT WAYNE | .59 | <.001 | 5.02 |
| US-IN-515 | CINCINNATI | .58 | .01 | 4.51 |
| US-IN-527 | INDIANAPOLIS | .75 | <.001 | 2.61 |
| US-IN-582 | LAFAYETTE | .08 | .77 | 5.27 |
| US-KS-603 | JOPLIN-PITTSBURG | .66 | .01 | 7.34 |
| US-KS-616 | KANSAS CITY | .76 | <.001 | 3.33 |
| US-KS-638 | SAINT JOSEPH | -.68 | .02 | 6.23 |
| US-KY-564 | CHARLESTON-HUNTINGTON | .82 | <.001 | 8.63 |
| US-KY-736 | BOWLING GREEN | .37 | .14 | 7.91 |
| US-LA-622 | NEW ORLEANS | .33 | .13 | 31.05 |
| US-LA-628 | MONROE-EL DORADO | .75 | <.001 | 3.67 |
| US-LA-642 | LAFAYETTE | .25 | .35 | 7.48 |
| US-LA-643 | LAKE CHARLES | .27 | .31 | 4.45 |
| US-LA-644 | ALEXANDRIA | .46 | .09 | 4.01 |
| US-LA-716 | BATON ROUGE | .60 | .01 | 3.88 |
| US-MA-506 | BOSTON | .63 | <.001 | 5.19 |
| US-MA-521 | PROVIDENCE-NEW BEDFORD | .79 | <.001 | 3.25 |
| US-MD-512 | BALTIMORE | .45 | .04 | 3.02 |
| US-MD-576 | SALISBURY | .15 | .54 | 4.22 |
| US-ME-500 | PORTLAND-AUBURN | .78 | <.001 | 3.36 |
| US-ME-537 | BANGOR | .35 | .16 | 3.94 |
| US-MI-505 | DETROIT | .62 | <.001 | 10.04 |
| US-MI-513 | FLINT-SAGINAW-BAY CITY | .65 | <.001 | 3.19 |
| US-MI-551 | LANSING | .58 | <.001 | 4.44 |
| US-MI-553 | MARQUETTE | .18 | .63 | 5.36 |
| US-MI-563 | GRAND RAPIDS-KALAMAZOO-BATTLE CREEK | .87 | <.001 | 5.34 |
| US-MI-588 | SOUTH BEND-ELKHART | .38 | .14 | 3.85 |
| US-MN-676 | DULUTH-SUPERIOR | .83 | <.001 | 5.07 |
| US-MN-702 | LA CROSSE-EAU CLAIRE | .49 | .05 | 4.78 |
| US-MN-724 | FARGO-VALLEY CITY | .73 | <.001 | 4.56 |
| US-MO-604 | COLUMBIA-JEFFERSON CITY | .32 | .20 | 3.74 |
| US-MO-631 | OTTUMWA-KIRKSVILLE | .13 | .68 | 4.43 |
| US-MO-717 | QUINCY-HANNIBAL-KEOKUK | -.09 | .75 | 6.04 |
| US-MS-647 | GREENWOOD-GREENVILLE | .63 | <.001 | 3.20 |
| US-MS-673 | COLUMBUS-TUPELO-WEST POINT | .53 | .02 | 4.24 |
| US-MS-710 | HATTIESBURG-LAUREL | .24 | .27 | 7.80 |
| US-MS-718 | JACKSON | .77 | <.001 | 5.37 |
| US-MS-746 | BILOXI-GULFPORT | .22 | .38 | 7.12 |
| US-MT-687 | MINOT-BISMARCK-DICKINSON | .30 | .23 | 4.52 |
| US-MT-754 | BUTTE-BOZEMAN | .59 | <.001 | 3.64 |
| US-MT-755 | GREAT FALLS | -.01 | .97 | 4.84 |
| US-MT-756 | BILLINGS | .62 | <.001 | 4.73 |
| US-MT-766 | HELENA | .15 | .51 | 3.90 |
| US-NC-517 | CHARLOTTE | .77 | <.001 | 4.08 |
| US-NC-518 | GREENSBORO-HIGH POINT-WINSTON SALEM | .65 | <.001 | 3.83 |
| US-NC-545 | GREENVILLE-NEW BERN-WASHINGTON | .58 | <.001 | 4.08 |
| US-NC-567 | GREENVILLE-SPARTANBURG-ASHEVILLE | .73 | <.001 | 4.38 |
| US-NC-570 | MYRTLE BEACH-FLORENCE | .51 | .02 | 5.60 |
| US-NE-722 | LINCOLN-HASTINGS-KEARNEY | .32 | .21 | 4.34 |
| US-NE-740 | NORTH PLATTE | -.28 | .28 | 4.82 |
| US-NE-759 | CHEYENNE-SCOTTSBLUFF | .34 | .17 | 5.35 |
| US-NM-790 | ALBUQUERQUE-SANTA FE | .49 | .02 | 4.79 |
| US-NV-839 | LAS VEGAS | .41 | .05 | 3.36 |
| US-NY-501 | NEW YORK | .60 | <.001 | 14.20 |
| US-NY-502 | BINGHAMTON | .57 | <.001 | 2.66 |
| US-NY-514 | BUFFALO | .26 | .27 | 4.71 |
| US-NY-523 | BURLINGTON-PLATTSBURGH | .67 | <.001 | 3.54 |
| US-NY-526 | UTICA | .67 | <.001 | 3.41 |
| US-NY-532 | ALBANY-SCHENECTADY-TROY | .67 | <.001 | 1.98 |
| US-NY-538 | ROCHESTER | .46 | .04 | 3.62 |
| US-NY-549 | WATERTOWN | .44 | .08 | 3.39 |
| US-NY-555 | SYRACUSE | .72 | <.001 | 3.51 |
| US-NY-565 | ELMIRA | .50 | .07 | 3.35 |
| US-OH-510 | CLEVELAND-AKRON | .76 | <.001 | 3.70 |
| US-OH-535 | COLUMBUS, OH | .55 | .01 | 5.32 |
| US-OH-542 | DAYTON | .65 | <.001 | 6.55 |
| US-OH-547 | TOLEDO | .71 | <.001 | 3.33 |
| US-OH-558 | LIMA | .57 | .18 | 7.06 |
| US-OH-597 | PARKERSBURG | -.29 | .36 | 6.22 |
| US-OK-634 | AMARILLO | .24 | .40 | 9.19 |
| US-OK-650 | OKLAHOMA CITY | .77 | <.001 | 5.41 |
| US-OK-657 | SHERMAN-ADA | -.14 | .67 | 5.75 |
| US-OR-801 | EUGENE | .30 | .21 | 4.99 |
| US-OR-821 | BEND | .50 | .02 | 4.55 |
| US-PA-504 | PHILADELPHIA | .60 | <.001 | 3.64 |
| US-PA-516 | ERIE | .29 | .30 | 5.39 |
| US-PA-566 | HARRISBURG-LANCASTER-LEBANON-YORK | .63 | <.001 | 4.37 |
| US-PA-574 | JOHNSTOWN-ALTOONA | .38 | .17 | 5.98 |
| US-SC-519 | CHARLESTON | .15 | .55 | 3.68 |
| US-SC-546 | COLUMBIA | .53 | .01 | 4.61 |
| US-SD-725 | SIOUX FALLS (MITCHELL) | .69 | <.001 | 4.51 |
| US-SD-764 | RAPID CITY | .58 | .01 | 3.87 |
| US-TN-557 | KNOXVILLE | .74 | <.001 | 4.65 |
| US-TN-639 | JACKSON | .19 | .52 | 3.37 |
| US-TN-659 | NASHVILLE | .51 | .01 | 3.75 |
| US-TX-600 | CORPUS CHRISTI | -.16 | .64 | 6.20 |
| US-TX-612 | SHREVEPORT | .79 | <.001 | 2.20 |
| US-TX-618 | HOUSTON | .56 | .01 | 3.87 |
| US-TX-623 | DALLAS-FORT WORTH | .57 | <.001 | 4.00 |
| US-TX-633 | ODESSA-MIDLAND | .49 | .05 | 5.24 |
| US-TX-635 | AUSTIN | .36 | .10 | 4.18 |
| US-TX-636 | HARLINGEN-WESLACO-BROWNSVILLE | .29 | .27 | 5.53 |
| US-TX-641 | SAN ANTONIO | .28 | .22 | 3.89 |
| US-TX-651 | LUBBOCK | .51 | .03 | 4.73 |
| US-TX-661 | SAN ANGELO | -.13 | .71 | 7.72 |
| US-TX-662 | ABILENE-SWEETWATER | .57 | .03 | 5.32 |
| US-TX-692 | BEAUMONT-PORT ARTHUR | .07 | .84 | 8.04 |
| US-TX-709 | TYLER-LONGVIEW (LUFKIN-NACOGDOCHES) | .63 | <.001 | 5.69 |
| US-TX-749 | LAREDO | .08 | .77 | 5.69 |
| US-TX-765 | EL PASO | .51 | .02 | 6.10 |
| US-VA-544 | NORFOLK-PORTSMOUTH-NEWPORT NEWS | .75 | <.001 | 4.10 |
| US-VA-556 | RICHMOND-PETERSBURG | .44 | .04 | 5.64 |
| US-VA-559 | BLUEFIELD-BECKLEY-OAK HILL | .52 | .03 | 5.69 |
| US-VA-569 | HARRISONBURG | .27 | .22 | 5.76 |
| US-VA-573 | ROANOKE-LYNCHBURG | .53 | .05 | 6.70 |
| US-VA-584 | CHARLOTTESVILLE | .38 | .12 | 4.98 |
| US-WA-819 | SEATTLE-TACOMA | .46 | .03 | 2.70 |
| US-WA-820 | PORTLAND | .71 | <.001 | 3.20 |
| US-WI-613 | MINNEAPOLIS-SAINT PAUL | .66 | <.001 | 3.90 |
| US-WI-617 | MILWAUKEE | .56 | .01 | 2.27 |
| US-WI-658 | GREEN BAY-APPLETON | .32 | .14 | 4.20 |
| US-WI-669 | MADISON | .55 | .01 | 2.92 |
| US-WV-598 | CLARKSBURG-WESTON | .49 | .05 | 6.52 |
